# Where’s Your Phone? A Survey of Where Women Aged 15-40 Carry Their Smartphone and Related Risk Perception: A Survey and Pilot Study

**DOI:** 10.1371/journal.pone.0167996

**Published:** 2017-01-06

**Authors:** Mary Redmayne

**Affiliations:** School of Public Health and Preventive Medicine, Monash University, Melbourne, Victoria, Australia; University of Chicago, UNITED STATES

## Abstract

Smartphones are now owned by most young adults in many countries. Installed applications regularly update while the phone is in standby. If it is kept near the body, this can lead to considerably higher exposure to radiofrequency electromagnetic radiation than occurred without internet access. Very little is known about current smartphone carrying habits of young women. This survey used an online questionnaire to ask about smartphone location under several circumstances to inform the power calculation for a women’s health study. They were also asked about risk perceptions. Data was analysed using Pearson chi square. Three age categories were made: 15–20, 21–30, 31–40. Smartphones were generally kept on standby (96% by day, 83% at night). Of all participants, in the last week the most common locations of the phone when not in use or during passive use was off-body (86%), in the hand (58%), a skirt/trouser pocket (57%), or against the breast (15%). Pocket and near-the-breast storage were significant by age (χ^2^15.04, p = 0.001 and χ^2^10.96, p = 0.04, respectively), both positively influenced by the youngest group. The same influence lay in the association between holding the phone (χ^2^11.082, p = 0.004) and pocket-storage (χ^2^19.971, p<0.001) during passive use. For calls, 36.5% solely used the phone against the head. More than half kept the phone 20–50 cms from their head at night (53%), while 13% kept it closer than 20 cms. Many (36%) thought RF-EMR exposure was related to health problems while 16% did not. There was no relationship between thinking RF-EMR exposure causes health problems in general and carrying the phone against the upper or lower body (p = 0.69 and p = 0.212, respectively). However, calls with the phone against the head were positively related to perception of health risk (χ^2^ 6.695, p = 0.035). Our findings can be used in the power calculation for a case-control study.

## Introduction

Smartphones are now owned by more than three quarters of US residents aged 12 to 34 [1], and 25% of US 18 to 33 year-olds who own a mobile cannot recall the last time it was not within arm’s reach [2]. In Australia, ownership among those aged between 18 and 75 is 89% [3] and in the United Kingdom 88.5% of 16–34 year olds owned one by 2015 [4]. In all cases, the highest proportion of owners are those under the age of 44 [5].

Use of these devices for purposes other than phone calls is high. A recent Pew study found that text messaging, voice and video calls, internet use, and email functions are used frequently by most smartphone owners [6]. Other popular uses for under-30s include social networking and listening to music/podcasts [6]. Activities such as the last of these can be undertaken without holding the phone, referred to as passive use in this paper. For instance, it may be tucked into the clothing and used with ear-buds.

These new uses and user-options and the high level of dependence on staying in touch carry implications for levels of radiofrequency electromagnetic radiation (RF-EMR) exposure. This can be considerably higher than used to occur in standby mode.

First, smartphones have several antennae capable of concurrent operation. The phones are naturally required to comply with exposure Standards. However, the antennae are tested individually and the reliability and appropriateness of the newly developed formula used to account for total output for compliance has been questioned [7].

Second, increased exposure occurs when the phone is in standby due to background activity related to installed applications (apps). Apps use one of three techniques, pull, push, and long-polling, that run automatically in the background for regularly updating information [8]. This is in addition to the regular signal maintaining contact with the nearest base station that applied to earlier mobile phones also. The type of polling used depends upon the app. For instance, Twitter uses pull technology, transmitting to the server for updates, while iPhone’s Apple’s Push Notification Service (APNS) sends messages asking for updates, remaining active while awaiting a reply; Facebook uses long-polling on iPhone but pull technology on android phones [8]. If there is no response, then the app has “heartbeats”–small signal packages to check whether there is an answer yet. These will occur at timed or variable intervals and continue until there is an answer or until the server decides to cut the link due to a period of activity with no “heartbeat”. The outcome of this is that apps that have not been disabled continue working in the background, regularly transmitting for short periods.

There is not a consensus of opinion on whether low, ongoing exposure to RF-EMR has adverse health consequences, but several biological effects have been demonstrated. Previously, concern about possible associations of mobile phone exposure with cancer/tumours has focussed human studies largely on brain tumours, due to the highest exposures occurring when the phone is closest to the body and transmitting. This mostly used to apply when the phone was used against the ear for a phone call, but now that smartphones continue to transmit when not in active use, it is important that the occurrence of other types of tumours, or other adverse health outcomes, in locations adjacent to common storage places should also be researched. For instance, tumour/cancer types to consider could include melanoma, breast cancer, ovarian cancer, leukaemia, and colon cancer.

Concern has been expressed about carrying a smartphone tucked into the bra against the breast, although research on possible impacts is sparse. A case study of four young women with no family history or genetic predisposition for breast cancer reported unusual tumours directly under or around the edges of the women’s usual storage location for the phone [9]. Research exposing MCF-7 human breast cancer cells to 900, 1800, and 2100 MHz in vitro found excessive ROS production and induced apoptosis when the antenna was less than 10 cm away [10]. Elsewhere, apoptosis was also induced, and cell viability reduced, after short term exposure in human breast fibroblast cells exposed to 2.1 GHz CDMA signal [11]. This signal and the exposure level used for that study is typical of some smart phones such as Samsung S6.

Rather little is known about current smartphone carrying habits. A multi-country study done early in the smartphone era [12] found the most common places for women to carry their phone was in a handbag (61%), trouser or skirt pocket (16%), with the hand being the third most popular place (9%). Women’s clothing often has no pockets, and a bag is not always carried. This can mean that the phone is tucked into clothing and is therefore carried directly against the body. In 2007, it was found that phone storage location depended upon context [12]. For instance, storage location varied considerably between beach and city areas in coastal USA, and in some areas of India women rarely carried a handbag and therefore more often used a trouser pocket. More recently, students aged 10 to 13 years reported most commonly storing their phone in the front/side pocket of a skirt or trousers [13]. In that study, 3 (< 2% of girls) reported keeping it tucked in the bra and a further 3 hung it round the neck against the chest, both locations being self-nominated in the ‘other’ category.

The aims of the study were to find out where and how near to the body smartphones are carried and used by young women. Participants were mostly drawn from the Melbourne Region of Australia. Melbourne is a city of 4.35 million and is culturally diverse, with 58% having parent/s born overseas and approximately one third of households speaking two or more languages [14]. The main goal of this pilot study is to enable the necessary power calculation to design a cancer or tumour study, thereby ensuring that the numbers enrolled are sufficient to find an effect if there is one to be found. Our principal interest was breast cancer in women due to the observed habit of carrying the phone tucked into a bra directly against the breast, hence the study being confined to women.

A risk perception component asked about whether participants think RF-EMR exposure is related to any health problems, allowing a preliminary examination of whether phone-carrying habits were related to concern about possible adverse outcomes. Between 1.5% and 13.3% of populations report experiencing a variety of health problems which are attributed to being in the vicinity of certain electromagnetic fields [15]. These can be quite disabling and imply “both acute and chronic inflammatory processes, involving mainly skin and nervous, respiratory, cardiovascular, musculoskeletal, and gastrointestinal systems” [16]. These symptoms collectively and in the absence of otherwise diagnosed organic disease are known as electrohypersensitivity (EHS). This is only acknowledged as being due to RF-EMR exposure in very few countries including Austria and Sweden.

## Method

The survey method utilized a questionnaire which was completed by participants online using Survey Monkey®. Potential participants were invited from a broad range of socioeconomic backgrounds and from many walks of life (Table 1).

**Table 1:** Backgrounds of those invited to participate.

Organisations through which participants were invited	Invited	Accepted
Secondary schools	17 private 6 State	0 private 1 State
Universities	5	2
Social/sporting/religious organisations and cafés	11	5
Mobile phone retails outlets	4	4
RF-EMF Awareness Group	1	1
Upmarket and downmarket major department stores	2 (1 of each)	2
Supermarkets	2	1
Community newspaper	1	1

Organisations were approached to notify their members, students, customers about the study but were not obliged to let us know if they had done so. They were not themselves participants.

Those who were invited included members of one RF-EMR awareness group and, to offset introducing possible bias, the employees of several mobile/smartphone sales centres/shops. Invitations were issued via those in a senior position (eg school Principal; club Committees/Chair) by phone and email, or by directly approaching head of human resources or store managers in department stores and shops. Each was provided with an explanatory notice about the research project and research group, the ethics approval information, and a link to the online questionnaire. There was also a poster to put up on noticeboards, and a sample email to send to employees or students, or to put in newsletters. In the case of those contacted first by phone, further information and materials were only sent if interest was shown and this was agreed. Those contacted were asked to use the materials provided to let their employees or students know about the project. The organisations themselves were not participants and were not obliged to advise me about whether they chose to notify their staff, customers, congregation, members or students. Members of more organisations than those who formally accepted may therefore have taken part.

Participants could choose a time or times that suited them to complete the questionnaire within the collection period which ran from April to August 2015.

Potential participants read background information online. This included a statement saying that taking part constituted consent for us to use the data provided. The first half of the questionnaire had qualifying and duration of ownership questions, and then focused on where the phone was kept, both when in use and when not in use, day and night. Responses for the ‘last 7 days’ and ‘ever’ were sought. Imputed affirmatives were assigned to ‘ever’ categories of use where the participant ticked ‘last 7 days’ but left ‘ever’ unanswered. Location of the phone under the following daytime circumstances was sought: When phone is not in use i) indoors ii) outside or in a vehicle, and iii) during passive use (eg GPS/listening to music). Each of these questions had the following response categories: In your hand; in a pocket; at or below waist level; in a bag; a vehicle, or elsewhere not in your clothing; in a breast pocket or hanging against your chest; in your bra or fitted sports top; against your upper arm; tucked into your hijab; other.

When the phone is being used for calls: against your ear; carried in your clothing with headset, hands free or speakerphone; in your hand but away from your body with headset, hands free or speakerphone.

When phone is being used for other purposes (passive use): on your lap resting against your abdomen; on your lap away from your abdomen; on a cushion on your lap, away from your abdomen; on a nearby surface while lying on your front/side; in your hands away from your body and lap. Before the second half on risk perception, there was the following statement:

For your information: The technical name for the energy given out by mobile phones is Radio Frequency Electromagnetic Radiation (RF-EMR). It is produced and emitted by electronic devices including mobile phones and tablets when transmitting voice and data. If smartphone apps are enabled, they can also transmit when the phone is in standby.

Participants were then asked about whether they had known these devices emitted RF-EMR, even if they did not know its name.

The next question (Q14) asked, “Do you consider that exposure to radio-frequency electromagnetic energy causes or worsens any health problem?” For ethical reasons, those who replied ‘No’, were not asked any further risk perception questions, but were directed to the last two questions on specific age and postcode.

Remaining participants were asked to rank their perceived likelihood of health risk with relation to using and carrying the phone against the body and with relation to specific diseases and conditions. Further questions asked about personal health-related experiences from using a phone (if any), and their level of concern.

After completion, participants were provided with a short, typical manufacturer’s notice about compliance testing distance and recommended minimum use and carrying distance for a mobile phone, and thanked for their participation.

Socio-economic status was estimated based on the Government ranking system called SEIFA, using the Index of Relative Socio-Economic Advantage and Disadvantage (IRSAD). This index is based on a broad range of indicators of advantage and disadvantage using home postcodes [17].

Eligible participants were female, aged 15–40 and carried a smartphone with them at least one day weekly. We excluded those who answered all questions in the first half in <3 minutes or finished all questions in <5 minutes.

### Analysis

Three age categories were created for chi-square analysis, 15–20 years (n = 27), 21–30 years (n = 71), and 31–40 years (n = 78). There were 10 other participants who did not provide their specific age and 11 who did not complete the survey as far as the specific age question, which was at the end.

All analyses using Pearson chi-squared were based on use over the 7 days prior to answering the questionnaire.

Pearson chi-square analysis was conducted using SPSS 22. Yates Correction for Continuity was applied in the cases of 2x2 tests. Adjusted standardised residuals, each of which is a z-score, were used to judge where the major influence lay on statistically significant results. Where the table was bigger than 2x2 and there was more than one significant z-score we observed where there was no influence on a significant outcome. The effect size was evaluated using either the phi coefficient or Cramer’s V, as appropriate.

A sensitivity analysis was carried out to assess the effect of excluding from the risk perception questions those who said “No” to the first risk perception question (Q14). The sensitivity analysis assumed that had those people been asked questions about their perception of risk in relation to where the phone was stored or used and specific diseases they would have said “No” or “Probably not”.

The study was approved by the Monash University Research Ethics Committee. No data identifying participants’ names, or their place of employment or study, or their phone numbers or home address, were collected.

## Results

### Participant profile

Of 228 respondents, 31 (13.6%) were excluded (15 were ineligible, 9 only answered the qualifying questions, and 7 answered too fast for answers to be reliable), leaving 197 participants.

Six percent did not respond to any risk perception questions; 16% of the others responded ‘No’ to the first one and were therefore directed to the last two questions of the questionnaire.

**Fig 1:**
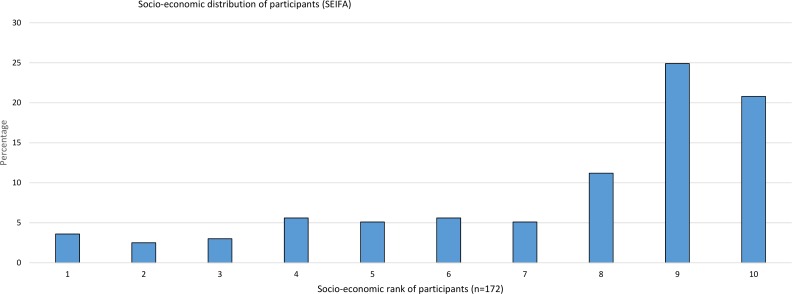
Socio-economic distribution of participants.

Socio-economic background of participants was right skewed (Fig 1) which is also the case for the population of Melbourne. The SEIFA rating of the residential area where participants lived (grouped as SEIFA 1–4, 5–8, 9–10) had no statistical significance with overall perception of risk from exposure to RF-EMR (χ^2^ 8.95, p = 0.062).

### Phone locations

The phone location results are reported in the following order: Location for calls; mode and location for other active use; mode and location for passive use; mode and location when phone is not in use by day both indoor and outside or in a vehicle; mode and distance of the phone during sleep.

#### Location for phone calls

**Fig 2:**
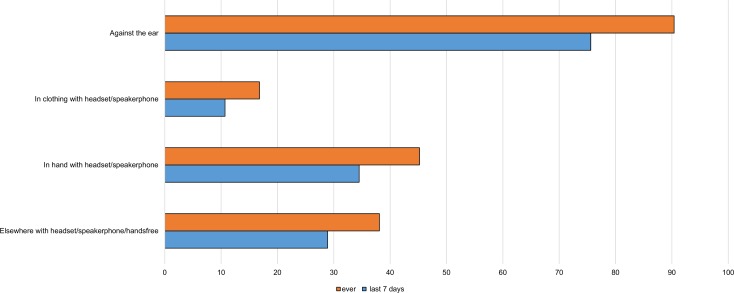
Location of phone during calls.

Most women held the phone against the ear when making or receiving calls (Fig 2). In the last week, 36.5% of all participants had only used the phone against the head when using it for calls, although 76% had done so at some time in that period.

#### Mode and location for other active purposes

The one most common location for the phone when being used recently online, seated or lying down, was in the hands, away from the body (79%); this was increasingly common with increasing age. There were insufficient numbers in some other categories for a valid chi square test. However some observations are worth mentioning as it may indicate differing levels of risk awareness in different age groups. A majority of the 4.5% of women who usually used the phone with it resting against the abdomen on the lap were aged over 30. On the other hand, of the 3% who usually rested the phone on a cushion on the lap, away from the body, most were under 21 and none was over 31 years. Most of the 5.6% who generally rested the phone on a nearby surface while using it lying down were aged 21–30.

#### Mode and location for passive use

Almost everyone (96%) kept the phone on standby during the day.

**Fig 3:**
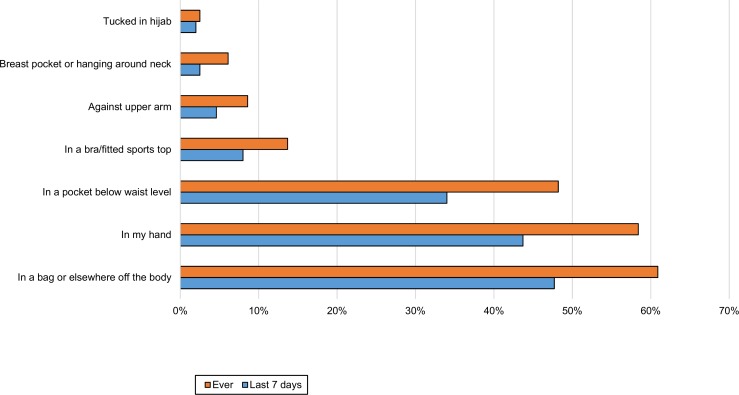
Location of phone during passive use (n = 197). There was a significant association with age for passive use in the hand (χ^2^ 11.082, p = 0.004, Phi 0.273) and in a pocket (χ^2^ 19.971, p<0.001, Phi 0.366). In both cases, the significance lay with a positive influence from the youngest group.

Participants were asked where their phone was when it was being used passively, such as for GPS, listening to music/radio/podcast online or streaming. Only ten (5%) responders said they did not use the phone this way. The most common locations in the last week were: not on the person (48%), in the hand (44%) or in a pocket below waist level (34%) (Fig 3).

#### Mode and location when not in use by day

**Fig 4:**
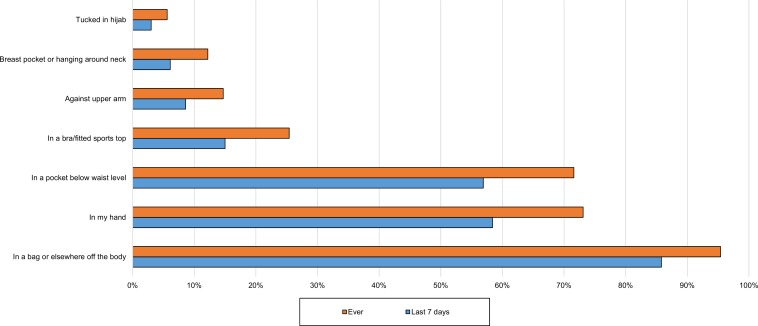
Location of phone for all daytime storage when not in use and during passive use (n = 197). This is the combined data from 3 questions: daytime phone location when indoors and not using the phone; when outdoors or in a vehicle and not using the phone; and when using the phone passively.

Overall, the most common location for the phone during the day, for all except active use, was in a bag or somewhere else off the body (Fig 4).

**Fig 5:**
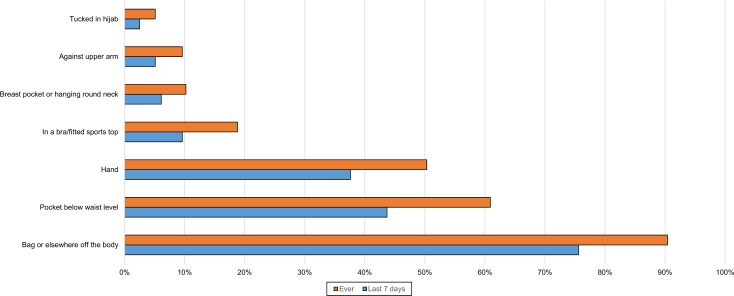
Location of phone for indoor daytime storage (n = 197).

**Fig 6:**
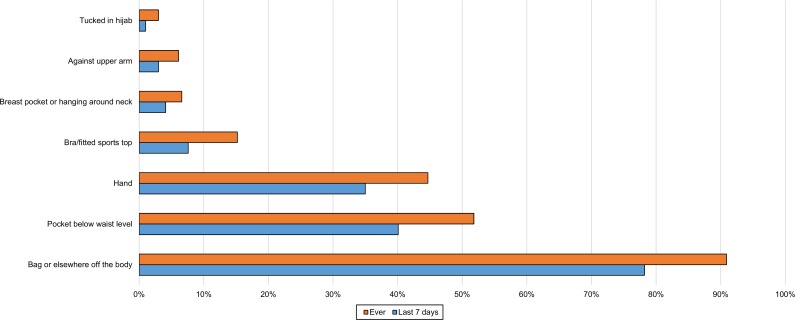
Location of phone for outdoor and in vehicle storage (n = 197).

**Fig 7:**
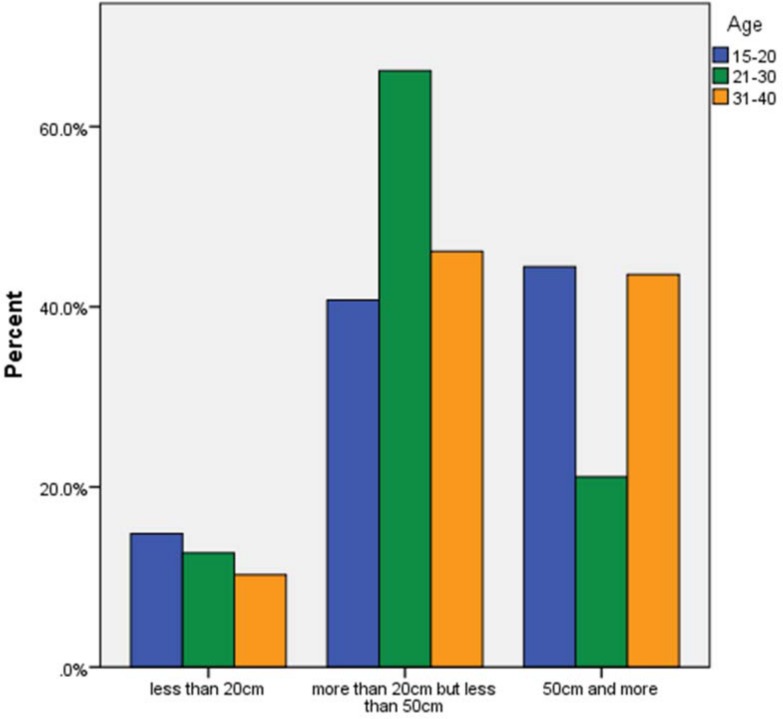
Distance of smartphone at night, by age. Participants were asked, “How far from your head is your phone at night? Tick the one that applies most often.” Most young adults kept their phone within reach at night.

Participants were asked to report all locations they used. Some of these were favoured more than others depending on whether women were indoors (Fig 5), outdoors or in a vehicle (Fig 6) or using the phone passively (Fig 3). A trouser or skirt pocket was the favoured on-body location when the phone was not in use (Figs 6 and 7), but the hand was narrowly favoured over the pocket for passive use (Fig 4).

Many (72%) had at some time stored the phone in a pocket below waist level and 57% had done so over the last week (Fig 4). Recent storage in the pocket below waist level was significant by age group, with more of the youngest group than expected likely to store their phone there (χ^2^ 15.04, p = 0.001, Cramer’s V 0.299). A quarter (25%) had at some time stored the phone tucked in their bra or a close fitting sports top, while 15% had done so during the last week (Fig 4). Again, it was significant by age group (χ^2^ 10.96, p = 0.04, Cramer’s V 0.255), there being a positive relationship with the youngest group. Most reported a few storage locations for each of these places, but in the last week 2.5% had only kept it in a pocket and 1% had only kept it in a bra when indoors.

Most of those who sometimes stored the phone hanging against the chest or in a breast pocket also used the bra or sports top for storage. A small group reported carrying their phone tucked into a hijab (Figs 3–6).

#### Mode and distance when sleeping

At night, 83% had their phone in standby mode, 7% flight mode and 10% turned it off.

More than half (53%) kept the phone between 20 and 50 cms from their head at night, while 13% kept it closer than 20 cms, most likely under, on or beside the pillow. Almost 90% of those who kept the phone closer than 20cms at night, had it in standby. Likewise, 91% who kept the phone 20-50cms at night had it on standby. Most (79%) of those who turned it off, had it >50cm away.

The 21–30 year olds were the least likely to have their phone more than 50cm away at night, and most likely to have it within arm's reach (20 to 50 cms) (χ^2^ 10.45, p = 0.033, Cramer’s V 0.172).

The distance of the phone at night was related to age (χ^2^ 10.45, p = 0.033, Cramer's V 0.172) (Fig 7). The strongest association was in the young adults being significantly unlikely to keep the phone more than 50cm from their head at night. Only 15% of the youngest group kept the phone within 20 cms of the head.

### Risk perception, behaviour and health

Only 5% of participants were unaware prior to doing the survey that smartphones and other wireless communication devices emit electromagnetic energy. The 21–30 year-old age group were best informed in this respect.

**Fig 8:**
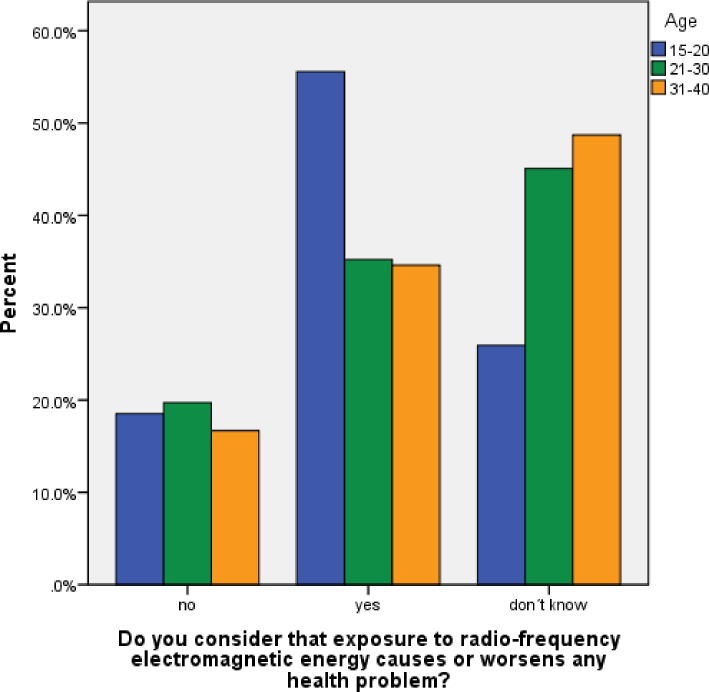
Perceived risk from RF-EMR (Q14), by age. All participants were asked, “Do you consider that exposure to radio-frequency electromagnetic energy causes or worsens any health problem?”

All participants were asked whether they considered that exposure to radio-frequency electromagnetic energy causes or worsens and health problem (Q14) (Fig 8).

Statistically, perceived health risk from RF-EMR did not vary by age (χ^2^5.187, df = 4, p = 0.269), although there was a tendency for uncertainty regarding risk (‘don’t know’ response) to increase with age (26%, 45%, 49%, respectively), and more than half (56%) of the youngest group thought RF-EMR exposure caused or probably caused or worsened health problems (Fig 8).

There was also no statistically significant relationship between thinking that RF-EMR exposure causes or worsens any health problem in general (Q14), and where the phone was carried: either against the upper body or against the lower body (p = 0.69 and p = 0.212, respectively).

However, an association did exist between perceived health risk (Q14) and whether or not the phone was used against the head for calls (χ^2^ 6.695, p = 0.035, Cramer’s V 0.202). This small size effect (Cramer's V 0.202) was due to a positive relationship between not using the phone against the ear and considering that doing so carried a risk.

Perceived risk did not impact on the daytime phone mode. However, there was a significant association between perceived health risk and the mode of the phone at night (χ^2^ 7.795, p = 0.02, Cramer's V 0.205). This small effect size was due to a significant positive relationship between those perceiving a risk and turning their phone off or putting it in flight mode at night.

The distance of the phone at night had a medium to strong relationship with perception of risk (χ^2^ 15.627, p<0.001, Cramer’s V 0.282). However, this was not influenced by those who kept it within 20cm, but was due to those who kept it >50cm away and off or in flight-mode and those who kept it between 20 and 50 cms away and in standby mode.

The 17% negative responders to Q14 were not asked further risk perception questions. The age breakdown was 15.5% aged 15–20, 44% aged 21–30, and 40.5% aged 31–40.

Of all those who said they considered that RF-EMR exposure does cause or worsen health problems (Q14), eleven subsequently gave solely negative responses to risk perception questions on specific disease and well-being outcomes.

There was a very strong positive relationship between considering RF-EMR causes or worsens any aspect of health and being fairly or very concerned about it (χ^2^ 37.459, p<0.001, df = 2, Cramer’s V 0.501). The level of concern was even stronger in a sensitivity test in which those who were excluded after saying they had no concern about health effects were added to the ‘no’ or ‘not very’ concerned group (χ^2^ 86.802, p<0.001, df = 4, Cramer’s V 0.490).

**Fig 9:**
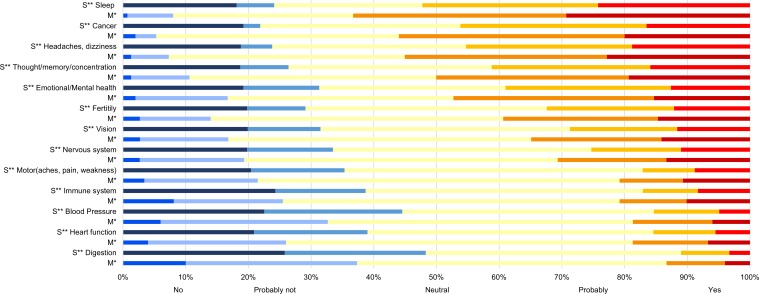
Perceived likelihood of specific outcomes from RF-EMR exposure. Participants were asked “Do you consider that exposure to radiofrequency electromagnetic radiation casus or worsens human health problems with respect to:” Specific end point were listed. The sensitivity and main dataset results are alternated for ready comparison.* Main dataset. Those who did not perceive a health risk at Q14 were not asked about their perception of risk for specific outcomes and are therefore excluded here (Q17) (n = 150). ** Sensitivity dataset. This included those who responded negatively to Q14; it assumes their responses would have been “No” (n = 182). Dark blues responded ‘No’, light blues responded ‘Probably not’, light yellow were not sure, orange responded ‘Probably’, red responded ‘Yes’.

Regarding specific disease or well-being outcomes, effects on digestion, blood pressure, and the immune system were of least concern, while, of most concern, were perceived links to quality of sleep; headaches and dizziness; and cancer (Fig 9)

### Perception and behaviour

#### Sensitivity analysis

As reported above, there was no statistically significant relationship between thinking that RF-EMR exposure causes or worsens any health problem generally speaking (Q14) and where the phone was carried: either against the upper body or against the lower body (p = 0.69 and p = 0.212, respectively). However, at a more personal level, perceived risk of storing or using the phone against the upper body was related to whether participants chose to do that (Table 2). The main data was checked with sensitivity tests. Excluding those who said they did not think RF-EMR caused or worsened any health problem did not affect the statistical significance of perceived risk in many cases, but did so in some (Table 2).

**Table 2:** Main analysis and sensitivity analysis to examine the effect of excluding those whose initial response to perceived health risk from exposure to RF-EMR was ‘No’.

	Main analysis ^a^			Sensitivity analysis^a^		
Question	N	χ^2^ (degrees of freedom) *p* [Cramer’s V]	Source of effect from highest z-score	N	Χ^2^ (degrees of freedom) *p* [Cramer’s V]	Source of effect from highest z-score when significant
*Perceived risk of* ***carrying*** *phone against body/head (Q15) vs behaviour (MP stored against upper body)*	145	χ^2^ **11.224 (2) *p* = 0.004 [0.278]**	Greatest effect from not concerned and carrying it there	185	χ^2^ **6.277 (2) *p* = 0.043 [0.184]**	Greatest effect from not being sure and not carrying it there
*Perceived risk of* ***using*** *phone against body/head (Q16) vs behaviour (MP stored against upper body)*	145	χ^2^ **9.912 (2) *p =* 0.007 [0.261]**	Same as above	185	χ^2^ 4.365 (2) *p* = 0.113 [0.154]	Not significant
*Perceived risk of* ***carrying*** *phone against body/head (Q15) vs behaviour (MP stored in pocket below waist)*	145	χ^2^ 0.340 (2) *p* = 0.843 [0.048]	Not significant	185	χ^2^ 3.096 (2) p = 0.213 [0.129]	Not significant
*Perceived risk of* ***using*** *phone against body/head (Q16) vs behaviour (MP stored in pocket below waist)*	145	χ^2^ 1.107 (2) *p* = 0.575 [0.087]	Not significant	185	χ^2^ 3.695 (2) *p* = 0.158 [0.141]	Not significant

There was a statistically significant association between carrying the phone against the upper body tucked in a bra or fitted sports top and perceived risk in doing so. This was not the case for storing or using the phone in a pocket and perceiving it as risky.

^a^ Those who considered RF-EMR exposure did not cause or worsen any health effect were not asked further risk perception questions for ethical reasons and were therefore excluded from the main analysis. The sensitivity analysis included them with an assigned negative response.

Carrying the phone against the upper body was not related to perception of risk of any specific disease outcomes (S1 Table), whereas carrying the phone against the lower body was perceived as risky for problems with digestion; the immune system; the nervous system; thought, memory and concentration; and emotional and mental health e.g. anxiety, mood, and depression (Table 3).

**Table 3:** Perceived risk of specific health problems from RF-EMR vs behaviour (storing phone in pocket below waist or not).

Perceived risk of health problems from RF-EMR (Q14) vs behaviour (storing phone in pocket below waist or not^a^)				
	Main analysis^b^		Sensitivity analysis^b^	
Health problem	N	χ^2^ (degrees of freedom) *p* [Cramer’s V]^c^	N	χ^2^ (degrees of freedom) *p* [Phi]^d^
*Digestion*	142	**8.924 (0.012)**	182	**10.668 (0.005)**
*Immune system*	142	**7.402 (0.025)**	182	**10.511 (0.005)**
*Nervous system*	142	4.285 (0.117)	182	**8.129 (0.017)**
*Cognition (thinking/memory/concentration)*	142	4.556 (0.102)	182	**8.034 (0.018)**
*Emotional/mental health (anxiety*, *mood*, *depression)*	142	4.736 (0.094)	181	**7.589 (0.022)**
*Heart*	142	2.790 (0.248)	182	5.928 (0.052)
*Blood pressure*	142	2.042 (0.360)	182	5.750 (0.056)
*Sleep*	142	3.058 (0.217)	182	5.595 (0.061)
*Aches*, *pains*, *weakness*	142	2.386 (0.303)	181	4.931 (0.085)
*Vision*	142	1.466 (0.480)	181	4.862 (0.088)
*Cancer*	142	1.092 (0.579)	182	4.538 (0.103)
*Headache*	142	0.680 (0.712)	182	2.457 (0.293)
*Fertility*	142	0.085 (0.958)	181	1.185 (0.553)

^a^Stored and/or used passively in a pocket below waist level.

^b^Those who considered RF-EMR exposure did not cause or worsen any health effect were not asked further risk perception questions for ethical reasons and were therefore excluded from the main analysis. The sensitivity analysis included them with an assigned negative response.

^c^2degrees of freedom

^d^1 degree of freedom.

Main analysis significance of perceived risk related to pocket carrying for digestion and the immune system lay in those thinking there was a risk and not keeping their phone in a lower pocket.

Sensitivity analysis significance for the immune system and nervous system lay in those thinking no/probably not a risk and keeping the smartphone in a lower pocket. Digestion significance was not influenced by the ‘not sure’ category. Both the significance of perceived risk to thinking/memory/concentration and emotional/mental health had statistically more than expected of those who were unsure refraining from keeping their phone in a lower pocket.

#### Health experiences attributed to mobile phones

Participants (n = 182) were asked if they had experienced any health problems because of using a smartphone; 88% (n = 149) had not, 3% no longer had that problem, and 9% still did. Specific symptoms experienced by participants and attributed to using a mobile phone included headaches (3%); sore or warm ear (1%); ringing in the ears and dizziness (1.6%); nausea (1%); tingling or shaking fingers (5%); heart palpitations, vision problems, difficulty concentrating, fuzzy thinking, sore thumb when texting on an older style phone, and cramps in a previously injured and permanently bent finger (0.6% each).

Two participants (1%) reported being electrohypersensitive but did not name specific symptoms. Only one participant (0.06%) reported having had a tumour or cancer (thyroid) and three others (1.6%) declined to answer.

There was just one experienced symptom specifically reported regarding storing a phone in clothing: “I used to always carry my phone in my left pants pocket, until I realised a few years back the constant pain in my abdomen near there disappeared when I didn't have my phone on me.”

#### Description of those excluded from the study

We only have age information for 18 (58%) of the 31 who were excluded/not eligible. Of these, 5 (28%) in the youngest group were underage, 5 (28%) were aged 21–30, and one (5%) was in the eldest group. Seven (39%) were either more than 40 or not female and were asked no further questions. The remaining eleven answered Q14 on risk perception. The five youngest (45%) all said 'no' or 'yes'. The others all said 'not sure'. This tendency of being sure, one way or the other, in the younger group, and less so in the older participants, was reflected in the study.

## Discussion

Many smartphone applications use pull or polling technology, and long polling, to keep updated with the latest available material, be it a social networking message, email, GPS position, or an advertisement about a clothing sale [18] and apps can update every 30 seconds. This means that for those who have several active applications such as social networks, messaging, marketing, GPS, and news, the phone is likely to transmit much of the time it is in standby. Each time, all the phone’s functions and apps are activated, not only the relevant app. Participants’ phones were usually left in standby.

The most common place for the phone when not in use was off the body, however, a large majority of women in our study regularly carried the phone in a trouser or skirt pocket (57% in the last week and almost three quarters had done so) and very similar numbers regularly carried it in their hand.

A surprisingly large proportion of women had carried their phone close to or against the breast recently (15%), including a third of the ≤ 20 age group. A quarter of women had ever done so. This is the necessary information for planning the proposed breast cancer study and would provide the same assistance for the study of a variety of other endpoints.

Even in standby and flight mode, the Central Processing Unit continues functioning. Induced electrical coupling occurs with close proximity for exposures at 450 MHz and lower [19]. This frequency or lower is used in iPhones by the graphics processing unit or central processing unit. These frequencies penetrate the body further than the higher carrier frequencies used for calls and internet.

There is some evidence that perception of risk may have impacted on behaviour to some extent such as whether or not to use the phone against the head when making calls, since those who were concerned about it were significantly less likely to use it that way.

The picture was more complex though for perception of risk for specific carrying locations versus behaviour versus perception of the risk of specific diseases. For instance, there was an association between perceived risk of carrying a phone in the bra and actually carrying it there, but there was a dissociation between that action and perceived risk of specific disease outcomes.

Concern over effects of RF-EMR on digestion and the immune system were the only specific diseases with a clear statistically significant relationship driven by those who chose not to keep their phone in a pocket. In the sensitivity analysis, other significant associations were found for nervous system complaints, thinking/memory/concentration, and emotional/mental health (anxiety; mood; depression). These symptoms are typical of those with electrohypersenstivity. However, other typical electrohypersensitivity symptoms were not statistically associated with phone storage against the breast or in a lower pocket (complaints such as heart function, vision, and motor problems such as aches, pains and weakness), so this should be interpreted with caution.

Interestingly, there seemed to be some dissociation between the three health problems that a majority were concerned about (sleep, cancer, headaches/dizziness) (Fig 9) and behaviour in terms of whether the phone was stored in either a pocket or by the breast (Tables 3 and S1).

We asked participants whether they attributed any health problems they experienced to use of a mobile phone. Although the survey focus was RF-EMR, this does not establish what aspect of the use they believe caused the problems. There are several possible explanations such as unusual posture or prolonged skin contact with the phone surface. Some symptoms may result from conviction that RF-EMR exposure is going to cause problems. Research has demonstrated this sometimes occurs although this explanation does not fit with the typically reported order of symptom onset first followed later by causal attribution [20]. Exposure to the phone’s RF-EMR emissions cannot be discounted as a cause and common, measurable changes in bio-chemistry have been demonstrated in many people reporting electrohypersensitivity [16, 21].

This study provides a considerable amount of new information on which parts of the body are most exposed to RF-EMR when smartphones are not being used, as well as during passive and active use. It would have been more helpful had we asked for information about storage location when people are outside separately from when in the car. Knowing duration of exposure in any one location would also be of help.

The ethical choice not to pursue further risk questions with those who did not perceive any risk impacted somewhat on analysis of possible links between perception of risk and behaviour. However, a useful amount of new information was gained using the one risk question (Q14) that all participants were asked. Future studies on smartphone storage could usefully include the use of an application in participants’ smartphones to measure actual use and/or exposure providing they agree not to lend their phone (or borrow others) during an agreed study period.

## Conclusions

The information collected in this study can be used in the power calculation for the number of people needed for a case-control study. Several such studies may be called for as so many people carry smartphones against the body where they continue to transmit. More than three quarters of participants had carried their phone in a skirt or trouser pocket, which places the phone adjacent to the skin of the lower abdomen or buttock sitting over or near the colon and near reproductive organs, and a quarter of them had carried it tucked into clothing against the breast. Overall, there was not a perception of risk from exposure to RF-EMFs generally, although when specific types of illness and/or specific on-body storage locations were analysed, it was apparent there was a significant concern about carrying and using a smartphone against the body/head (although this behaviour was common). There was also a significant relationship between digestion and the immune system, and carrying the phone in a lower pocket. The sensitivity analysis indicated concern about keeping the phone in a pocket and several typical electrohypersensitivity symptoms.

## Supporting Information

S1 Table Perceived risk of specific health problems from RF-EMR vs behaviour (storing phone against upper body or not). Unlike general perception of risk related to storing the phone by the breast and where it was actually stored, this table indicates that perception of risk of specific disease outcomes were not related to carrying the phone there.
^a^Stored and/or used passively in the bra, sports top, breast pocket, or on a neck holder.
^b^Those who considered RF-EMR exposure did not cause or worsen any health effect were not asked further risk perception questions for ethical reasons and were therefore excluded from the main analysis. The sensitivity analysis included them with an assigned negative response.
(TIF)
