# Supplementary figures and images for: Where’s Your Phone? A Survey of Where Women Aged 15-40 Carry Their Smartphone and Related Risk Perception: A Survey and Pilot Study
Source: PLoS One. 2017 Jan 6;12(1):e0167996. doi: 10.1371/journal.pone.0167996 (PMC5218506; doi:10.1371/journal.pone.0167996)

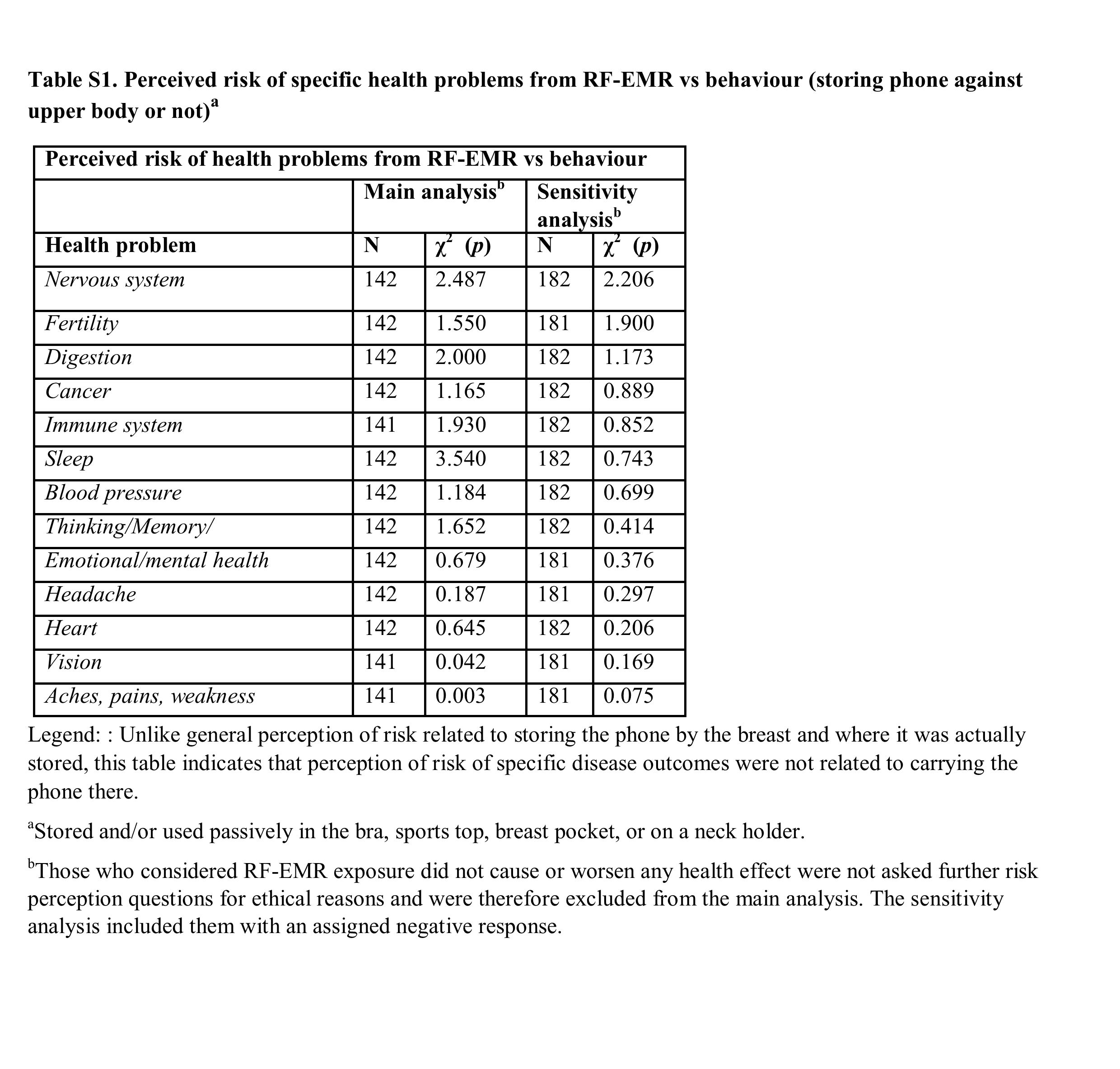

Supplement: S1 Table — Unlike general perception of risk related to storing the phone by the breast and where it was actually stored, this table indicates that perception of risk of specific disease outcomes were not related to carrying the phone there. aStored and/or used passively in the bra, sports top, breast pocket, or on a neck holder. bThose who considered RF-EMR exposure did not cause or worsen any health effect were not asked further risk perception questions for ethical reasons and were therefore excluded from the main analysis. The sensitivity analysis included them with an assigned negative response. (TIF) [file pone.0167996.s001.tif]
